# Application of Big Data and Artificial Intelligence in COVID-19 Prevention, Diagnosis, Treatment and Management Decisions in China

**DOI:** 10.1007/s10916-021-01757-0

**Published:** 2021-07-24

**Authors:** Jiancheng Dong, Huiqun Wu, Dong Zhou, Kaixiang Li, Yuanpeng Zhang, Hanzhen Ji, Zhuang Tong, Shuai Lou, Zhangsuo Liu

**Affiliations:** 1 Medical Big Data Research Center, The First Affiliated Hospital of Zhengzhou University, Zhengzhou, China; 2 Department of Medical Informatics, Medical School of Nantong University, Nantong, China; 3 Department of Health Technology and Informatics, The Hong Kong Polytechnical University, Hong Kong, China; 4 The Third Affiliated Hospital of Nantong University, Nantong, China; 5 Jiangsu Zhongkang Software Co, Ltd, Nantong, China

**Keywords:** Big data, Artificial intelligence, COVID-19, Deep learning, Epidemic prevention and control

## Abstract

COVID-19, caused by severe acute respiratory syndrome coronavirus 2 (SARS-CoV-2), spread rapidly and affected most of the world since its outbreak in Wuhan, China, which presents a major challenge to the emergency response mechanism for sudden public health events and epidemic prevention and control in all countries. In the face of the severe situation of epidemic prevention and control and the arduous task of social management, the tremendous power of science and technology in prevention and control has emerged. The new generation of information technology, represented by big data and artificial intelligence (AI) technology, has been widely used in the prevention, diagnosis, treatment and management of COVID-19 as an important basic support. Although the technology has developed, there are still challenges with respect to epidemic surveillance, accurate prevention and control, effective diagnosis and treatment, and timely judgement. The prevention and control of sudden infectious diseases usually depend on the control of infection sources, interruption of transmission channels and vaccine development. Big data and AI are effective technologies to identify the source of infection and have an irreplaceable role in distinguishing close contacts and suspicious populations. Advanced computational analysis is beneficial to accelerate the speed of vaccine research and development and to improve the quality of vaccines. AI provides support in automatically processing relevant data from medical images and clinical features, tests and examination findings; predicting disease progression and prognosis; and even recommending treatment plans and strategies. This paper reviews the application of big data and AI in the COVID-19 prevention, diagnosis, treatment and management decisions in China to explain how to apply big data and AI technology to address the common problems in the COVID-19 pandemic. Although the findings regarding the application of big data and AI technologies in sudden public health events lack validation of repeatability and universality, current studies in China have shown that the application of big data and AI is feasible in response to the COVID-19 pandemic. These studies concluded that the application of big data and AI technology can contribute to prevention, diagnosis, treatment and management decision making regarding sudden public health events in the future.

## Introduction

**Fig. 1:**
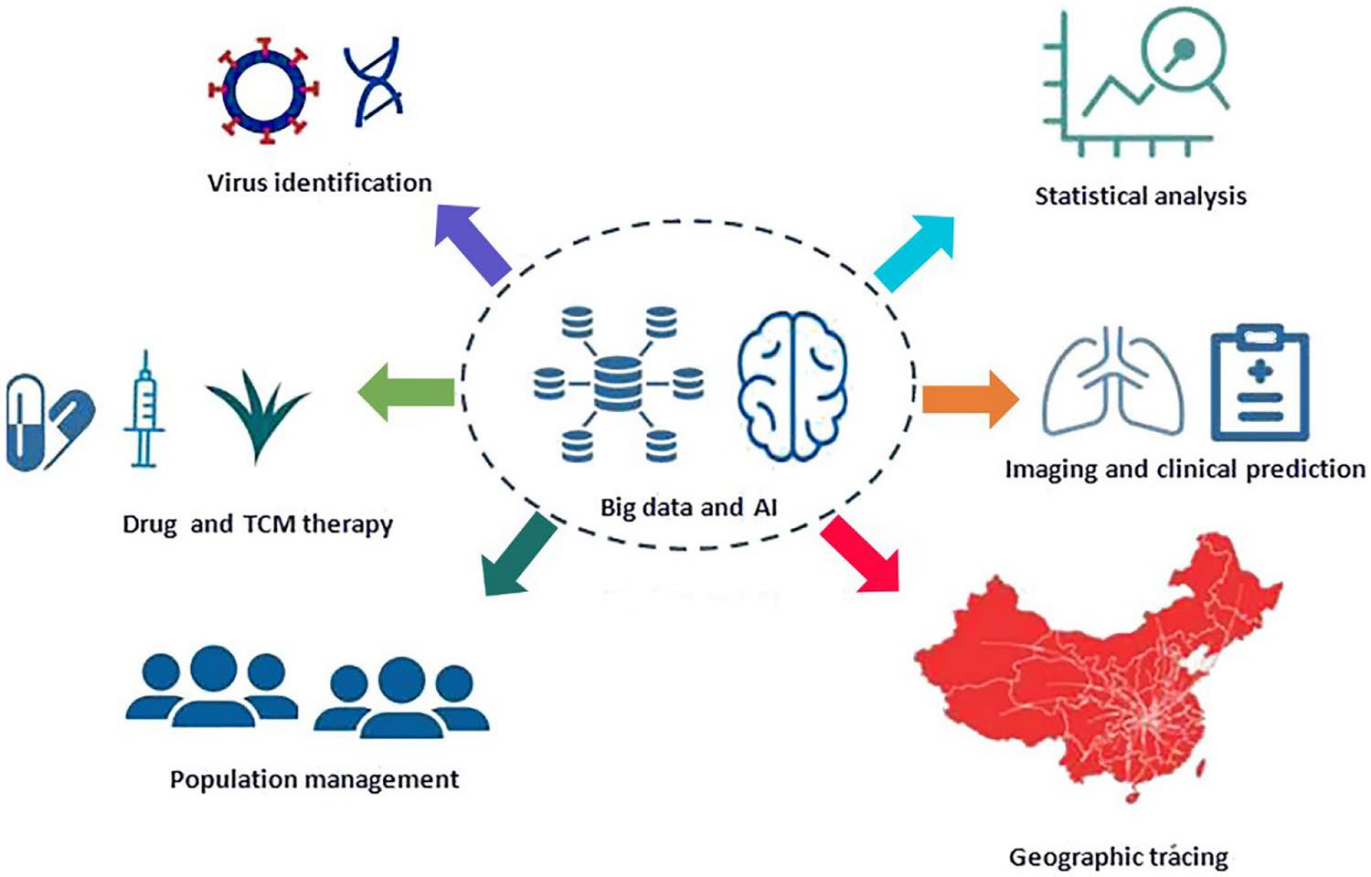
Diagram of summarized domains in COVID-19 pandemic prevention and control in China

The coronavirus disease 2019 (COVID-19) pandemic caused by severe acute respiratory syndrome coronavirus 2 (SARS-CoV-2) is raging throughout the world. As of February 21, 2021, the cumulative confirmed cases reported to the World Health Organization (WHO) exceeded 110,763,000, the cumulative number of deaths was 2,455,000, and the COVID-19 pandemic was still the most severe global health emergency [1]. Upon the outbreak of COVID-19, China was the first country to share SARS-CoV-2 genome sequence data with the WHO and the international community [2]. With the help of scientific technologies and resources, the Chinese government adopted the most comprehensive, stringent and thorough prevention and control measures in an attempt to bring the virus under control and finally achieved an initial triumph. The new generation of information technology, represented by big data and artificial intelligence (AI), has been widely applied in epidemic prevention and control, diagnosis and treatment as well as management decisions and has played valuable roles in the fight against the COVID-19 pandemic. The COVID-19 pandemic presents a unique background to the emergency response, diagnosis and treatment of sudden public health events in that the COVID-19 pandemic has had different forms along with the evolution of the disease, and it has also required taking the ever-changing epidemic situation, the treatment conditions of patients and their response to various treatment decisions into consideration. Although science and technology have developed, there are still challenges with respect to epidemic surveillance, accurate prevention and control, effective diagnosis and treatment, and timely judgement. Epidemic assessment usually relies on the real-time monitoring of disease control systems, and big data and related technologies promote multi-dimensional data integration and epidemiological analysis. In particular, the qualitative interpretation of AI-powered lung imaging includes the description of lung imaging changes over time, the prediction of clinical manifestations and outcomes, and the assessment of the effects of disease and treatment on systemic organs [3–6]. This paper reviews the current situation, challenges and prospects of the application of big data and AI in the prevention, diagnosis, treatment and management of COVID-19 in China, with the intention of explaining how to apply big data and AI technology to address common problems in the COVID-19 pandemic. Although the findings regarding the application of big data and AI technologies in sudden public health events lack validation of repeatability and universality, ongoing and completed studies in China have shown that the application of big data and AI is feasible in response to the COVID-19 pandemic. In this review, we summarized the application of big data and AI technology in different domains with efforts made in China, aiming to provide a reference for prevention, diagnosis, treatment and management decision making regarding sudden public health events in the future (Fig. 1).

## Big data analysis in COVID-19 prevention and control

In public health emergencies, massive daily data on the internet can be used to track epidemic progression and public concerns and to monitor crises, predict epidemic tendencies in advance and provide early warnings [7]. Peng et al. [8] created an analysis framework concerning the five dimensions of information, including the epidemic itself and the medical, governmental, public and media responses. According to early information on COVID-19 in China, they divided the framework into five stages, namely, the latent stage, first outbreak stage, first plateau stage, second outbreak stage and second plateau stage, thus providing an important reference for the early prevention and control of COVID-19 in different regions and populations. In the early stage of COVID-19, Zhao et al. [4] tracked the spread of SARS-CoV-2 based on big data and used the traffic flow data from Baidu Map to carry out linear correlation analysis between the population who left Wuhan and real-time-updated data of confirmed cases of China. The results showed that except for Hubei Province, the population of each province and the population exported from Wuhan contributed to 50% and 10% of the cumulative number of confirmed cases in each province, respectively; that is, 50% of the confirmed cases in each city were related to the local population, and 10% of the confirmed cases were related to the population exported from Wuhan. At the early stage of the outbreak in China, the amount of transmission was closely related to the number of people who left Wuhan. At the beginning of the spread of COVID-19, there was a high correlation (0.71) between the number of confirmed infection cases and air passengers from Wuhan. With the implementation of traffic control and other restricted population movement measures in Hubei Province, the correlation coefficient quickly dropped to 0.56 and then gradually decreased, which proved that Wuhan was the source of COVID-19 in China. Infected cases (71%) in other cities were related to the population who left Wuhan, and the spread trend of COVID-19 varied in different epidemic stages. Although these methods for analysing epidemic situations using internet data often refer to the paradigms of big data application, this model ignores the large amount of information that can be extracted by traditional statistical methods, and its relative value as an independent epidemic monitor is worth further discussion [9].

**Fig. 2:**
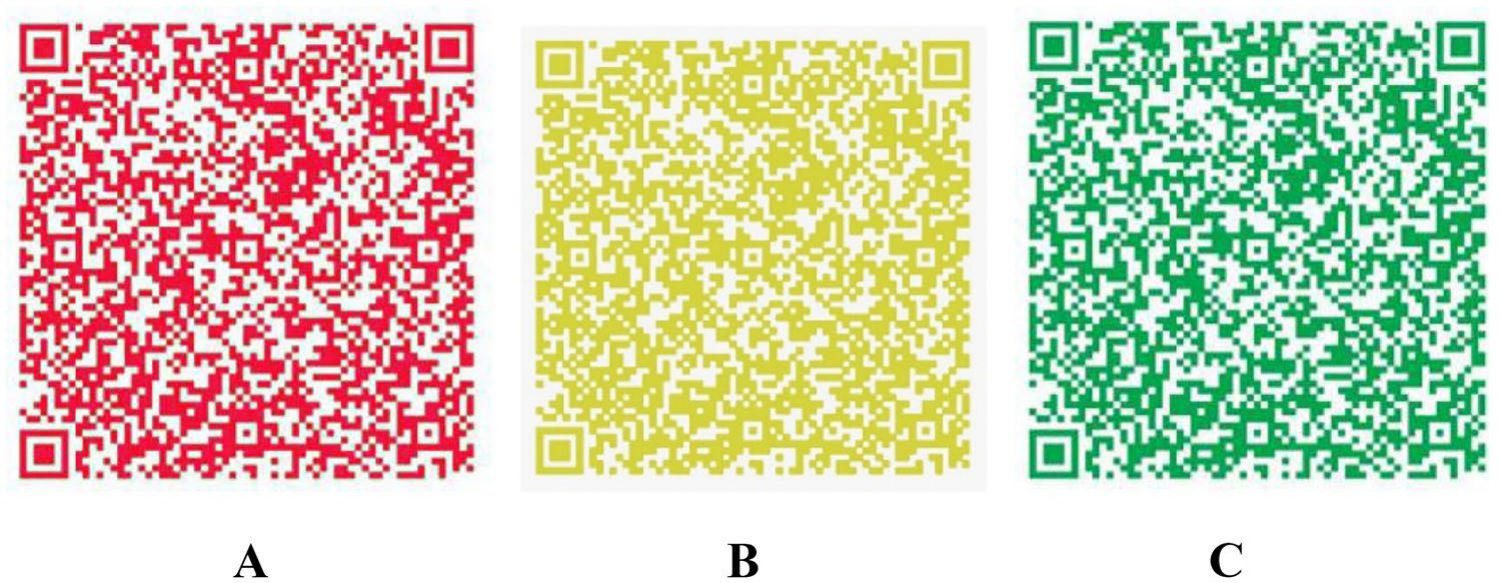
The definition of populations represented by different colour health codes. A: Red code: including confirmed cases, suspected cases, asymptomatic cases, persons who had close contact with confirmed cases, and persons under medical observation. B: Yellow code: including fellow travellers who have no close contact with confirmed cases, persons registered in fever clinics, persons with discomfort such as fever, fatigue, cough, diarrhoea and conjunctival congestion or persons who left a high-risk epidemic area in the past 14 days. C: Green code: indicating that the health status of the holder is basically normal, without discomfort, and that they are allowed to move around and resume work and production.

The complexity of big data analysis lies in the integration of different types of information to transform a large amount of data into operational knowledge for precision medicine and decision makers [10]. Technically, it is possible to build a big data platform to store and integrate high-capacity and high-diversity biological, clinical, environmental and lifestyle information related to health status collected from individuals and population at one or more time points for real-time demand to be beneficial for the public and the government [11]. At the early stage of the epidemic, Zhejiang Province was the first province to initiate the first-level public health emergency response, following Hubei Province. To cut off the spread of the epidemic caused by the movement of people, Yuhang district in Hangzhou city took the lead in implementing closed management of communities. On the evening of the same day, Alibaba Group organized a relevant technical team to urgently develop an early version of the “Yuhang Green Code”, which tried to declare personal information and travel data independently through mobile phones instead of paper proof and on-site information registration. Meanwhile, authenticity verification was performed in the background database to determine whether the person had a contact history of an epidemic area or with high-risk personnel. Just one week later, this system was launched on Alibaba’s Alipay platform and officially named the “Health QR Code”, which was then applied in many provinces and cities. By the beginning of March 2020, the National Government Service Platform was connected to the Alipay Health Code, which began to be applied nationwide [12, 13]. The key to the health code is that the verification of data authenticity needs the support of different databases, including the data of confirmed and suspected cases from the National Health Commissions, close contact data from the Ministry of Transport, floating population data from public security, and mobile phone roaming data from telecommunications operators. Data integration and intelligent analysis through the big data platform can be used to distinguish whether an individual is a COVID-19-infected patient, close contact, fellow traveller without close contact, person registered in a fever clinic or person with normal health condition, and finally, two-dimensional health code images with three different possible colours (red, yellow and green) are generated, to divide all of the floating population into three categories (Fig. 2), which is convenient for governments at all levels, institutions and communities to take corresponding prevention, control and management measures [14, 15]. The implementation requires trans-department collaboration to break down the barriers between organizations, the support of big data and AI technology, and epidemic management and unified organization from the government. With the continuous improvement in its application, the health code has played an irreplaceable role in normalized epidemic prevention and control, work resumption and economic recovery in China [16] (Fig. 2).

## AI in COVID-19 diagnosis and treatment

The COVID-19 pandemic spread around China rapidly. After the first case was reported in Wuhan, COVID-19 spread to 31 provinces and cities within one month, and the number of confirmed cases reported to the Chinese Center for Disease Control and Prevention (CDC) reached 44,672 [17]. Along with the high infection rate and high mortality rate of COVID-19, medical workers lack a comprehensive understanding of the clinical characteristics of COVID-19 and face challenges in diagnosis and treatment decision making. In the early stage of the outbreak, clinicians collected various data of COVID-19 patients for analysis and judgement, explored diagnosis and treatment rules, and exchanged clinical experience. They organized and formulated the first edition of the Diagnosis and Treatment Protocol for COVID-19 Patients and revised it five times within one month under the leadership of the National Health Commission [18, 19]. On the basis of accumulating data, medical workers analysed the clinical characteristics, CT manifestations, and treatment methods and efficacy in different types of patients with the help of statistics, big data and AI technology, [20–26] which provided justification for the revision of Diagnosis and Treatment Protocol for COVID-19 Patients. Currently, the eighth edition of the Diagnosis and Treatment Protocol for COVID-19 Patients has been officially released, which has played an active guiding role in the diagnosis and treatment of COVID-19 in China [27].

Applications of AI in the medical and health fields have made exciting progress, especially the increasing application of AI in disease diagnosis. Some AI applications can even propose treatment regimens or treatment suggestions [28, 29]. Zhang et al. [30] developed a lung lesion segmentation model and a diagnosis analysis model with an AI system by using a large CT database (China Consortium of Chest CT Image Investigation, CC-CCII) from 3,777 patients. For the classification model, 361,221 CT images from 2,246 patients, including 752 novel coronavirus pneumonia (NCP) patients, 797 common pneumonia patients, and 697 normal control patients, were used for training. This AI system’s diagnostic prediction performance was tested in one retrospective cohort and three prospective pilot studies. In addition, 456 hospitalized patients with comprehensive CT images and clinical outcome information were subjected to prognosis prediction and survival analysis and the establishment of a clinical prognosis estimation model. This AI system can diagnose NCP and differentiate it from other common pneumonia and normal control conditions, which can assist physicians and radiologists in performing a quick preliminary examination and/or screening, shorten the diagnostic workflow and patient waiting time, reduce the overall workload of radiologists, and respond more effectively and quickly than medical personnel under emergency situations. It has been noted that this AI system can also identify important clinical markers that correlate with NCP lesion properties. Together with clinical data, this AI system is able to provide accurate clinical prognosis that can aid clinicians in considering appropriate early clinical management and allocating resources appropriately. Currently, this AI system is available globally to assist clinicians in combatting COVID-19.

**Fig. 3:**
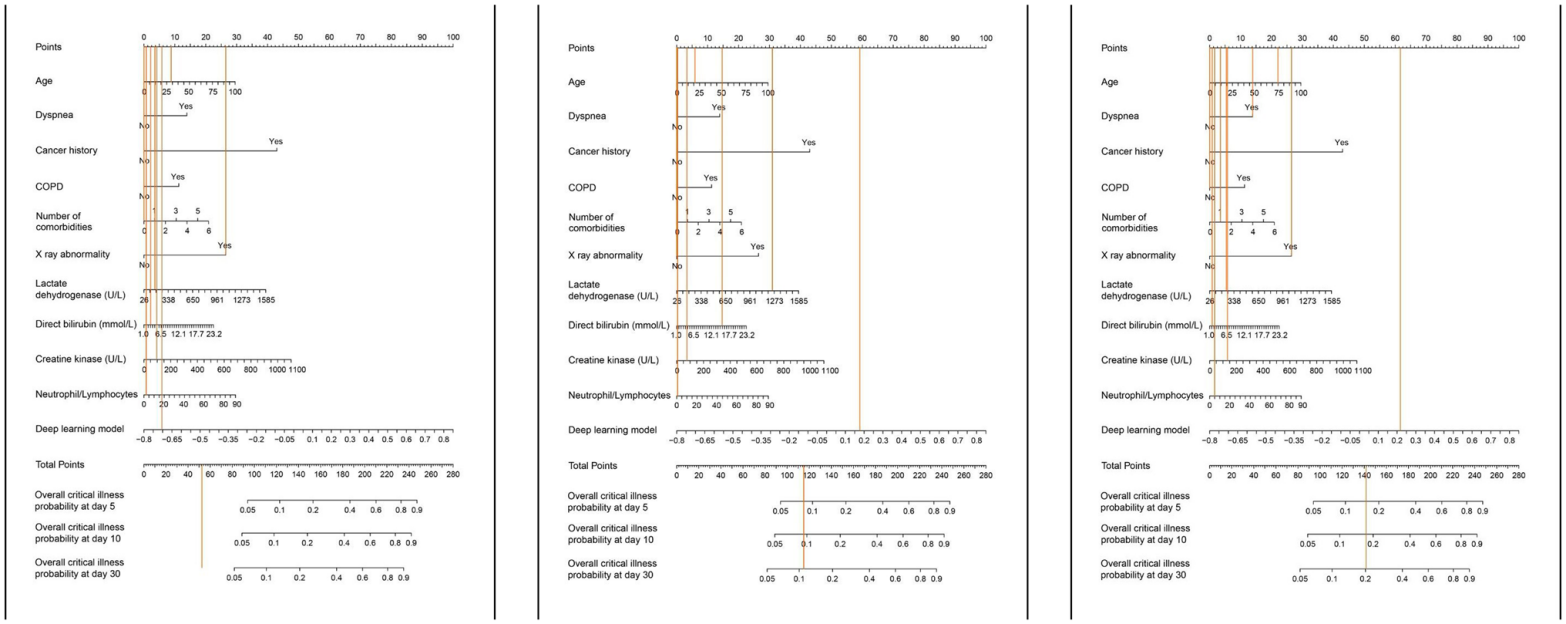
The nomogram prediction results of three different severe cases by the COVID-19 model developed by Zhong’s team [33]

Existing studies have shown that approximately 6.5% of COVID-19 patients and 20% of hospitalized COVID-19 patients have severe symptoms and need intensive care, which results in a mortality rate of 49% and consumes many medical resources [31, 32]. Therefore, early identification of patients with high-risk critical illness and timely disease intervention are significant to predict disease progression and allocate medical resources efficiently. A top Chinese physician, Zhong Nanshan, [33] cooperated with the Tencent-affiliated artificial intelligence laboratory (AI Lab) and integrated deep learning technology with a traditional Cox model to perform survival analysis on the nonlinear effect of clinical covariates with the aim of predicting the clinical outcome of COVID-19 patients. They established a retrospective cohort of COVID-19 patients in China and a model training cohort of 1,590 COVID-19 patients (131 severe patients) from 575 hospitals. Ten clinical features with statistically significant risk ratios were identified from 74 clinical features related to patients with critical illnesses by machine learning, and finally, a COVID-19 prediction model was developed (Fig. 3).

The AI prediction model for COVID-19 disease progression can predict the probability of critical illness in COVID-19 patients after 5, 10 and 30 days, which enables more rational risk stratification and corresponding diagnosis and treatment for patients. Additionally, three sets of data from different geographical regions and health resources (940 cases in Wuhan city, 380 cases in Hubei Province except Wuhan city and 73 cases in Guangdong Province) were tested for external validation. The results showed that the prediction model had good universality. This prediction model is open-sourced and can be optimized with increasing application data to improve its accuracy. [33].

Traditional Chinese medicine (TCM), characterized by dialectical treatment, has a long history in treating infectious diseases and has made indelible contributions to the health of the Chinese nation [34]. After obtaining relevant syndrome data of patients, evidence-based diagnosis and treatment can be conducted [34–37]. At the early stage of the COVID-19 pandemic in China, the TCM field responded to the call of the Chinese government, [38] thoroughly investigated the epidemic area of Wuhan and explored the TCM regimen for clinical diagnosis and treatment and community-based prevention and control in response to new and sudden infectious diseases [39–42]. The app-based data acquisition system for confirmed COVID-19 cases was developed for the first time, and medical staff could collect data on syndromes of confirmed cases in hospitals in Wuhan through mobile phones. After data collection, cleaning and privacy protection, the data were analysed by the Chinese Supercomputing Center according to the principles of TCM, and evidence for NCP belonging to a category of “damp toxin epidemic” in TCM was obtaine [43, 44]. According to this analysis, the rules of TCM treatment were clarified, effective TCM formulas were established, and finally, “three formulas and three medicines” were determined [45, 46]. By using big data analysis and artificial intelligence technology, the action mechanism of Qingfeipaidu decoction [47–49] was analysed, and a network molecular pharmacology study confirmed the active ingredients in this herbal formula, which laid a foundation for the development of related new drugs [46, 50, 51]. After pharmacological studies and first-line clinical trials in Wuhan, it was finally confirmed that this herbal formula could control the pulmonary inflammation of COVID-19 patients and inhibit the COVID-19 virus [48]. This herbal formula has already been applied as a new drug in China and received clinical trial approval from the US Food and Drug Administration on July 16, 2020. Currently, the study team is working with US universities to launch clinical trials in the US in the future [52]. Evidence has proven that big data and AI technologies are of great value in promoting the development of TCM diagnosis and treatment, Chinese medicine research and development, and clinical evaluation [53–55]. Several treatment regimens for the combination of Chinese and Western medicine to combat COVID-19 have been proposed, and TCM treatment regimens were described in the third through eighth editions of the Diagnosis and Treatment Protocol for COVID-19 Patients in China [56, 57]. Under the support of clinical big data technology, the TCM team in Wuhan has also created a “Wuchang model” based on the “TCM universal formula + community + internet”, which greatly reduced the incidence of COVID-19 in high-risk populations, relieved the disease of mild patients, and moved the focus of the anti-epidemic work into the community, which has bought precious time for the restoration of the medical system [58, 59].

The first novel coronavirus strain was successfully isolated [60] on January 24, 2020, which means that the vaccine strain could be cultured to prepare a vaccin [61, 62]. With the improvement of science and technology, based on the accumulating data on severe acute respiratory syndrome (SARS) and Middle East respiratory syndrome (MERS) and the support and promotion of relevant national policies, five COVID-19 vaccines have initiated human trials in China, and advanced AI technology has played an important role in accelerating the speed and improving the quality of vaccine research and development [63, 64].

First, in the discovery stage of vaccines, it often takes several years to perform manual analysis. However, machine learning can complete data analysis and matching of potential pharmacological relationships, screen drug action targets from massive data, find biomarkers, simulate synthesis of lead compounds, and conduct multiple rounds of analysis on the structure–activity relationship of vaccines, which greatly improve the research speed and effectiveness of vaccines [65, 66]. Second, the application of AI in pharmacokinetics, safety pharmacology and toxicology, preparation development and phase I, II and III clinical trials accelerates the research speed and improves the quality and effectiveness [67–69]. For example, in pharmacokinetic studies, a neural network system has been used to simulate the absorption, distribution, metabolism and excretion in animals and produce the optimized pharmacological structure to improve the accuracy and speed of experiments [70–72]. In toxicology studies, a deep learning method was used to analyse the toxicity mechanism, mode, pathway and effect of various vaccines, which could reduce the pressure of clinical trials and save study cost [65, 73]. Third, AI can promote intelligent manufacturing for large-scale vaccine production. Based on the deep integration of new-generation information and communication technology and advanced manufacturing technology, a new production mode with functions featuring self-perception, self-learning, self-decision-making, self-implementation and self-adaption functions throughout production, management and service can greatly improve the production, quality and efficiency of vaccines and promote the rapid entry of vaccines into the market [74, 75]. Finally, AI can intelligently track and monitor the use of vaccines after marketing. A vaccine monitoring system for epidemic prevention can be established to realize remote, digital and dynamic tracking and monitoring and collect data for use in a timely manner, thus providing guidance for further research and development, production and application of vaccines [76–78].

## AI in epidemic prevention and control and management decisions

Immediately after the COVID-19 outbreak, the central authorities led by Chinese President Xi put people’s life and health first and fought to block the spread of the virus, putting forward the overall principles of supporting confidence, strengthening unity, ensuring science-based control and treatment, and implementing targeted measures [79]. After the COVID-19 virus database developed by the China National Center for Bioinformation (CNCB) was officially launched, the genome and variant information of the COVID-19 virus were released in time, [80] which strongly promoted international cooperation in epidemic prevention and control [81–83]. Based on the big data of the epidemic situation in Wuhan and the whole country, the water, land and air transportation in Wuhan were closed in time, and the government rallied 346 national medical teams, consisting of 42,600 medical workers and 925 public health professionals, to the immediate aid of Hubei Province and Wuhan city. [84].

In the process of epidemic prevention and control, the application of big data and AI provides support in timely research and judgement of the epidemic trend, epidemiological investigation, identification of every infected person, and close contact tracing and isolation [85, 86]. By establishing a large-scale cross-regional, cross-departmental, cross-industrial and cross-structural shared database to provide risk data for epidemic prevention and control in accordance with the law, accurately identify different risk populations, and predict epidemic risks in different regions, AI provides effective services for orderly movement of people and resumption of work and production [87, 88]. Through real-time 5G video conversation, epidemiological investigation teams in remote mountainous areas can interact with high-level experts on a platform thousands of kilometres away [89–92]. With the authorization of individual citizens, [93] the personal “Health QR Code” and “Communication Big Data Travel Card” have been promoted nationwide as certificates for personnel travel, resumption of work and education, daily life and access to public places. Traffic control and classified disposal are carried out based on the query results, and accurate identification, precise implementation and precise prevention and control are realized at different levels in different regions [94]. An “epidemic map” is drawn using big data technology, and the specific location, distance and number of people for epidemic transmission are indicated by using the name, address and location of the community members, which provides convenience for the public to prevent COVID-19 [95–97].

Six months after the WHO announced that the COVID-19 pandemic constituted a “public health emergency of international concern” on January 31, 2020, the WHO issued a statement for the third time on August 1, 2020, declaring that the COVID-19 pandemic was still a “public health emergency of international concern”. It is expected that the epidemic will last for a long time, and it is necessary for countries around the world to strengthen cooperation and take long-term response measures [98]. China’s successful experience in epidemic prevention and control has been recognized, learned from and applied by many other countries, and their fight against the virus has also achieved good results [99–101].

## Challenge and future

Although big data and AI have been successfully applied in the prevention and control of the COVID-19 pandemic, they still face obstacles and challenges before widespread clinical application [102]. With the global prevalence of COVID-19, the management of epidemic data and medical data has been a major obstacle to the development of intelligent prevention and control and clinical solutions [103, 104]. Big data is a necessary prerequisite for AI training [105]. Standardized data are particularly important in the field of health care, especially multi-region, multi-system and multi-source heterogeneous data [106]. Although epidemic data disclosure is an encouraging step forward, the research community has not yet reached a consensus on specific data sets, and there is a lack of shared and collaborative data sets that are proven to be universal, repeatable and standardized for sharing and collaboration. Governments, professional organizations and institutions at all levels should be encouraged to share validated data to support the development of AI algorithms [107]. Another obstacle is the interpretability of AI, the ability to question the reasons behind particular outcomes and the expectation of failure [108, 109]. With the emergence of deep learning and prediction models, applications must be constantly updated according to the use of real-world data (RWD) in training. The application of AI requires trained professionals, who are expensive in time and cost; [110, 111] however, it also raises concerns about personal privacy and data security [112, 113].

The ultimate goal of health care is to prevent and control diseases. Establishment of an accurate risk model is vital to guide the strategy of risk adjustment. Therefore, AI systems must meet strict clinical test requirements, which will become an important direction of future development [114, 115]. With the expanded sources of big data, the data sources are not limited to electronic medical records (EMRs) and electronic health records (EHRs). Currently, data from wearable devices, mobile phones, social media and others are also available [116, 117]. AI is very suitable for integrating parallel information flows from biology, the population and society to improve the prediction models and medical intervention for patient outcomes, including quality control and risk assessment [118, 119].

As the application potential of big data and AI in the medical field is increasingly confirmed, there are still many directions for the transition from current application to conventional clinical practice. For example, for medical image analysis, the accuracy and prediction ability of the AI method need to be significantly improved. To replace the workflow of clinicians, it needs to be proven that the effectiveness of the AI method is better than that of human experts in a control study [120]. In addition, the application of AI in monitoring health resources and outcomes may improve efficiency and reduce costs. Like any innovative technology, the development of big data and AI will certainly be beyond the imagination of humankind [121].

## Conclusion

On February 4, 2020, the Ministry of Industry and Information Technology of China released an initiative that called on giving full play to the utility of AI in the fight against the novel coronavirus outbreak [38] Facing the challenge of COVID-19, breakthroughs in the application of AI have been accelerated, and it has been again verified that AI technology has a profound effect on current medical health and human behaviour and will play a greater role in the fight against the COVID-19 pandemic and other events that may be faced in the future [122, 123]. At present, the main applications and trends are as follows. first, big data analysis will be more intelligent. It will be easier to find epidemics at the early stage, track close contacts, improve diagnosis and treatment efficiency, predict the possible evolution of viruses in the future and develop more effective and long-lasting vaccines by analysing massive and real-time data with machine learning and deep learning [124–126]. Second, epidemic prediction will be more accurate. Most AI algorithms are prediction oriented, and the unique skill of AI-assisted epidemiological research will be used to establish a system that can accurately predict when and where future outbreaks will occur and how human behaviours will change to improve the ability to detect and respond to epidemic risks [127, 128]. Third, detection and prevention of the pandemic will be automated. Computer vision technology will be used to screen individuals with COVID-19 symptoms such as fever, and facial recognition technology will be used to track the activities of individuals with symptoms in a crowd and inform relevant departments or managers of the statistical data and probability of virus transmission [129, 130].
